# Distal radial fractures heal by direct woven bone formation

**DOI:** 10.3109/17453674.2013.792769

**Published:** 2013-05-31

**Authors:** Per Aspenberg, Olof Sandberg

**Affiliations:** Department of Orthopedics, Clinical and Experimental Medicine, Faculty of Health Sciences, Linköping University, Linköping, Sweden.

## Abstract

**Background:**

Descriptions of fracture healing almost exclusively deal with shaft fractures and they often emphasize endochondral bone formation. In reality, most fractures occur in metaphyseal cancellous bone. Apart from a study of vertebral fractures, we have not found any histological description of cancellous bone healing in humans.

**Patients and methods:**

We studied histological biopsies from the central part of 12 distal radial fractures obtained during surgery 6–28 days after the injury, using routine hematoxylin and eosin staining.

**Results:**

New bone formation was seen in 6 cases. It was always in the form of fetal-like, disorganized woven bone. It seldom had contact with old trabeculae and appeared to have formed directly in the marrow. Cartilage was scarce or absent. The samples without bone formation showed only necrosis, scar, or old cancellous bone.

**Interpretation:**

The histology suggests that cells in the midst of the marrow respond to the trauma by direct formation of bone, independently of trabecular surfaces.

The main burden of fractures, in terms of absolute frequency, monetary loss, and loss of quality of life, comes from fractures in osteoporotic, cancellous bone. Despite this, our knowledge of fracture healing in cancellous bone is limited. More or less everything known about fracture healing biology is based on animal models of long bone shaft fractures. However, there may be differences in how cancellous and cortical fractures heal. Fractures in cancellous bone often heal without any external or periosteal callus. They engage a marrow rich in stromal cells or mesenchymal stem cells (MSCs) that can be drawn upon in a trauma-healing scenario. In contrast, cortical fractures usually occur in diaphyseal areas where the marrow is mostly fat with a limited content of MSCs, the contribution of which to healing may be minimal (Colnot et al. 2006). Thus, cortical fracture healing would have to rely more on cells drawn from the periosteum, other surrounding tissues (mainly muscle), and the blood. (For a comprehensive review of the sources of cells in fracture healing, see Schindeler et al. (2009)). Furthermore, MSCs residing in the metaphyseal part of rodent long bones have a higher rate of division and are more active in their interactions with immune cells, as compared to MSCs residing in the diaphysis (Siclari et al. 2012). The consequences of the differences in cell sources for the healing process are unknown.

The scarcity of knowledge about cancellous fracture healing is partly due to the lack of animal models. Long bone shafts of rodents can be easily broken, and the strength of the healing structure can be measured by bending tests. In contrast, mechanical testing of the healing of injured cancellous bone is complicated, and until the last decade no animal models for this were available. Moreover, the story of long bone healing being in part a recapitulation of endochondral formation during fetal development has strong appeal.

In this descriptive study, we used histology to examine fracture healing in cancellous bone. It is an old idea. More than 15 years ago, we took a single, very small biopsy from a distal radial fracture 12 days after injury. It showed woven bone, forming directly in the marrow compartment and not primarily on the surface of old trabeculae. In order to confirm this unpublished observation, we have now studied more biopsies, taken from patients with distal radial fractures 5–28 days after the fracture occurred.

## Patients and methods

12 patients (5 men) between the ages of 22 and 77 years were operated on with volar plating because of malposition of a distal radial fracture, with a delay of 5–28 days. The reasons for the delay varied. In most cases, an initially acceptable post-reduction position of the fracture had been lost at 1 week follow-up. In other cases, the surgery was delayed due to patients travelling, or for administrative reasons. There was no case of delay because of other medical conditions. Patients consented to a biopsy being taken, and the study was approved by the regional ethical board (#2011/131-31). The biopsies were taken by several surgeons who were not otherwise involved in the study. The surgeons were instructed to take a biopsy the size of a peppercorn or less from the center of the fracture. This was done using a small, sharp spoon or rongeur entered into the central region of the fracture via the volar fracture opening.

The biopsies were prepared with routine methods for decalcified paraffin histology, sectioned all through in 5-µm thick slices, and stained with hematoxylin and eosin. Qualitative inspection of the slides was performed, noting the occurrence of bleeding (hematoma), soft tissue necrosis, old bone trabeculae (with or without osteocytes), bone formation on the surface of old bone, bone formation without contact with old bone, and cartilage. The occurrence of bleeding, bone formation, and cartilage was graded from 1 to 3, where 1 meant some, 2 meant a lot, and 3 meant that the occurrence dominated the entire specimen. Due to a suggestion by one of Acta’s reviewers, we looked through the specimens once more and found 2 cases with minimal amounts of bone on a trabecular surface and noted that specifically (Table).

**Table:** 

Patient	Day	Bleeding	Soft tissue	Old	Old	Bone	Free, woven	Cartilage	
				necrosis	trabeculae	surviving	formation on	bone	
						osteocytes	trab. surface		
11	5	3	1	1	0	0	0	0	
8	7	1	0	1	1	0	0	0	
4	8	2	0	1	1	(1) **^a^**	0	0	
1	9	1	0	1	0	0	1	0	
5	10	2	1	1	1	0	0	0	
7	12	0	1	1	0	0	1	0	
10	12	0	1	1	0	0	0	1	
2	13	0	0	1	0	(1) **^a^**	3	0	
3	13	1	1	1	0	0	0	0	
6	14	0	0	1	0	0	3	1	
9	16	0	0	1	1	1	3	0	
12	28	0	0	0	0	0	3	1	

0 = none; 1 = some; 2 = moderate; 3 = predominating. **^a^** Parentheses indicate a few cells found during a second review of the slides.

## Results

**Figure 1.**
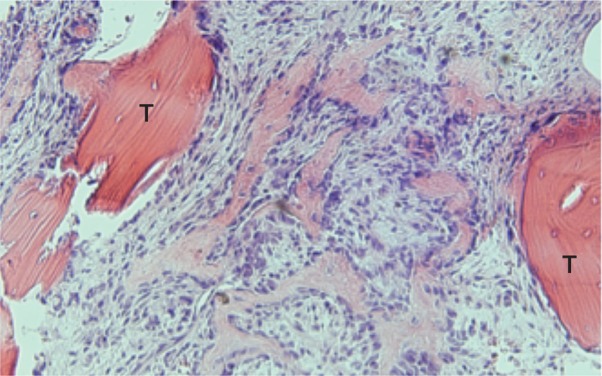
Membraneous bone formation at an early stage, between old necrotic trabeculae (T), 13 days after injury.

**Figure 2.**
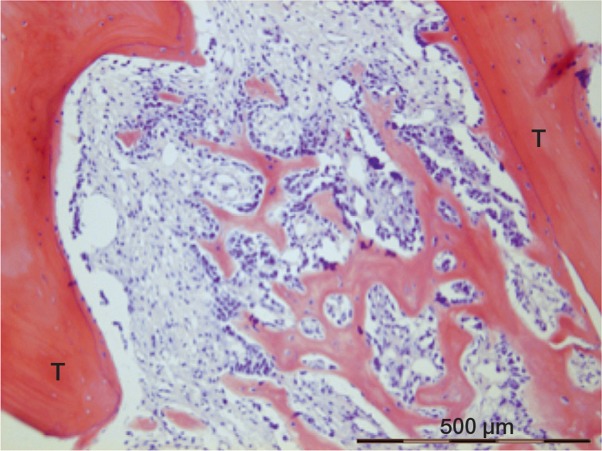
Formation of woven bone in the marrow cavity between 2 old trabeculae (T), in which superficial osteocytes have survived. Specimen from 16 days after injury.

**Figure 3.**
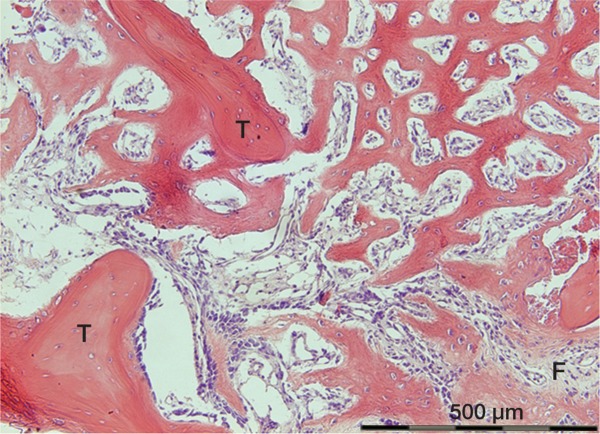
Woven bone in the marrow connecting to old trabeculae (T). Formation is still continuing at the lower right-hand corner (F). The same patient as in Figure 2.

All biopsies contained old bone trabeculae. 6 biopsies showed some form of new bone formation. In all these cases, the by far most predominant form of bone formation appeared as membranous ossification within the marrow space (Table and Figures 1–3). The new bone was loose and woven, with large rounded osteocytes. There was no obvious relation between this new-formed bone and the adjacent old trabeculae, although in some instances a thin layer of lamellar bone was seen on some trabeculae.

Of the 6 cases without new bone formation, all showed a hematoma or bleeding and 5 showed necrotic tissue. There were no signs of ongoing healing. However, osteocytes were still present in 3 of these cases.

Remnants of old dead bone with empty osteocyte lacunae were seen in 10 cases. New bone on the surface of these trabeculae was seen in 2 cases—in both cases close to membraneous ossification in the adjacent marrow.

**Figure 4.**
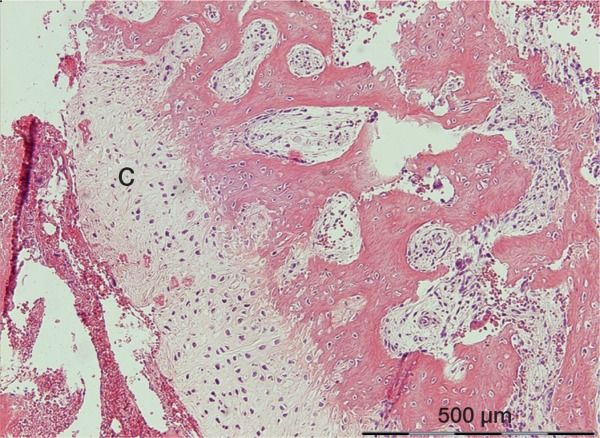
The most prominent occurrence of cartilage (C) in the study. It is located between woven bone and a necrotic hematoma. There appears to be a gradual transition from woven bone, via fibrocartilage, to hyaline cartilage.

Cartilage was seen in scant amounts in 3 cases, all dominated by new woven bone formation. The cartilage mainly occurred in relation to necrotic areas. A gradual transition from cartilage to woven bone could be seen, suggesting that a gradient in local conditions had governed tissue differentiation (Figure 4). Endochondral ossification was never seen, but the small amounts of cartilage observed are most likely prone to undergo this process in due time.

Bleeding and necrosis occurred mainly up to 10 days after injury. Woven bone was first seen after 9 days.

## Discussion

**Figure 5.**
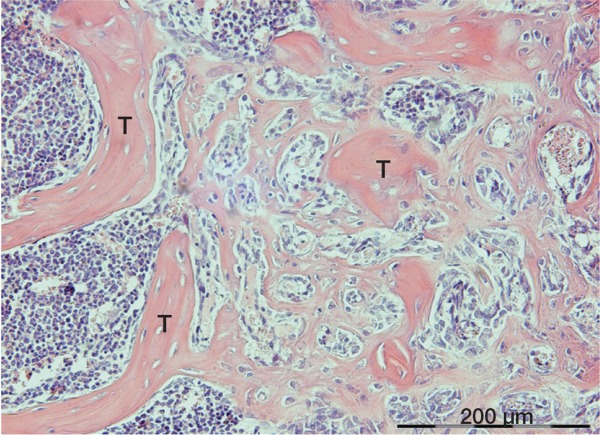
Woven bone formation among old trabeculae (T) in the proximal tibia of a mouse, 1 week after drilling a hole through the marrow. Bone formation appears to occur in the marrow compartment and to connect to old trabeculae, as for the human biopsies.

In all cases where new bone formation was detected, this occurred mainly by direct formation of fetal-type bone in the marrow during the second week after injury. Although small amounts of cartilage were seen in 3 cases, endochondral ossification appears to have played a minor role, if any. The new bone formation was mainly located in the midst of the marrow between trabeculae, or within soft callus-like tissue, with only minor contribution from the adjacent surfaces of old trabeculae. This observation suggests that stromal cells in the marrow may be a more important source of new bone than lining cells on trabecular surfaces. These results are in agreement with own unpublished findings in rodent models. Drill holes in metaphyseal bone in rats or mice show an inflammatory reaction followed by direct woven bone formation 7 days after injury, with a histological picture resembling those in our human biopsies (Figure 5).

Osteoconduction is a phenomenon whereby bone is thought to fill a defect by forming preferentially on certain surfaces. Dead bone trabeculae may offer ideal osteoconductive surfaces (Burchardt 1983). Still, only minute amounts of new bone formation were seen on such surfaces in our biopsies (Figures 1 and 2), suggesting that osteoconduction is not an important part of healing of these fractures. Also, studies of retrieved allografts show little evidence of osteoconduction (Enneking and Mindell 1991). Bone chamber experiments testing porous materials with suggested osteoconductive properties have also shown little or no powerful osteoconductive effect (Wang and Aspenberg 1996, Walschot et al. 2011).

There have been few published histological findings to describe cancellous fracture healing in humans. One study used biopsies taken from osteoporotic vertebral fractures (Diamond et al. 2007). The authors described fracture healing based on a predetermined model, dividing the process into 4 sequential phases, of which cartilage formation or “hyperosteoidosis” was the second. They reported not seeing any membranous bone formation, but their term hyperosteoidosis appears to represent direct ossification, which we regard as being synonymous with membranous ossification. It might correspond to the primitive woven bone that we report here. However, their biopsies were taken several weeks after the fracture whereas our biopsies were taken earlier. This makes comparisons difficult.

A major weakness of this study is its simple morphologic nature, which does not allow any tracing of the prehistory or origin of the cells observed. However, it appears evident that cells responsible for membranous ossification within a traumatized marrow compartment are derived from the same marrow. Although it may seem that we have kicked in an open door, there are no previously reported data on the histology of healing human fractures in cancellous bone outside the spine. Another weakness is that biopsies were not taken from any external callus. Thus, we cannot exclude predominant cartilage formation in this region, although we find this unlikely. In a previous study of the radiographs of 27 distal radial fractures, the mineralization of the external callus appeared to be even, and never showed any voids that could represent cartilaginous areas (Aspenberg and Johansson 2010).

It is not known what triggers bone formation in the marrow. Platelet activation and danger-associated molecular patterns (DAMPs) activate the inflammatory response that is thought to start healing in general (Lindemann et al. 2001, Bianchi 2007), but how this healing is directed to bone formation is unknown, although it should be related to the marrow milieu. DAMPs may consist of certain intracellular proteins that can activate the innate immune system when released or leaking from damaged cells into the extracellular environment. Although extravasated erythrocytes were common in the specimens, they were never seen in the areas with ongoing bone formation. This might suggest that bleeding is a less important signal, but too long a time may have passed since the injury for such observations to be valid.

In conclusion, the early healing response in distal radial fractures appears to involve activation of marrow cells, forming woven bone directly in the bone marrow, with no obvious need of cancellous bone surfaces for cell supply or osteoconduction.
